# Effect of gamma (γ-) radiation on the opto-structural and morphological properties of green synthesized BaO nanoparticles using *Moringa Oleifera* leaves

**DOI:** 10.1016/j.heliyon.2024.e26350

**Published:** 2024-02-13

**Authors:** Md Rabiul Islam, Sapan Kumar Sen, Arup Kumar, M.S. Islam, Md. Serajum Manir, Zannath Ara, M.D. Hossain, M.K. Alam

**Affiliations:** a Institute of Radiation and Polymer Technology, Atomic Energy Research Establishment, Bangladesh Atomic Energy Commission, Dhaka, 1349, Bangladesh; b Institute of Electronics, Atomic Energy Research Establishment, Bangladesh Atomic Energy Commission, Dhaka, 1349, Bangladesh; c Materials Science Division, Atomic Energy Center, Dhaka, Bangladesh Atomic Energy Commission, Dhaka, 1000, Bangladesh; d Department of Nanomaterials and Ceramic Engineering, Bangladesh University of Engineering and Technology, Dhaka, 1000, Bangladesh; e Department of Chemistry, University of Dhaka, Dhaka, 1000, Bangladesh; f Ministry of Labour and Employment, Government of the People's Republic of Bangladesh, Dhaka, 1000, Bangladesh; g Department of Physics, Sher-E-Bangla Nagar Adarsha Mohila College, Dhaka, 1207, Bangladesh; h Department of Physics, Bangladesh University of Engineering and Technology, Dhaka, 1000, Bangladesh

**Keywords:** BaO, Green synthesis, Gamma irradiation, *Moringa Oleifer*, Williamson-Hall method

## Abstract

In this current assessment, BaO synthesized from *Moringa Oleifera* leaves were irradiated using 0–75 kGy gamma radiation and investigated its physical impacts. The x-ray diffraction (XRD) data demonstrated the synthesis of tetragonal BaO, and no phase deviation was observed after irradiation. As doses are increased, the overall crystallite size were decreased due to an increase in defects and disorders. The tetragonal BaO was evident in Fourier transform infrared (FTIR) spectra prior to and following irradiation, while peak intensities and wavenumbers varied considerably. The as-prepared BaO showed a spherical shape morphology, and Field emission scanning electron microscopy (FESEM) indicated no vital deviations in it after irradiation. As irradiation shifts from 0 to 75 kGy, optical bandgap was increased from 4.55 to 4.93 eV, evaluated using Kubelka-Munk (K-M) equation from UV–vis–NIR spectrophotometer. Opto-electronic and photonic devices have challenges in extreme radiation conditions, such as space and nuclear environments. So, these assessments suggested that BaO can withstand high levels of gamma photon and could be a good option for photonic and optoelectronic instruments in an extreme gamma-ray exposed conditions.

## 1 Introduction

Nanomaterials (NMs) with the particle dimension at the nanometer scale (10^−9^ m) i. e ranging from 1 to 100 nm have special properties that are very different compare to their bulk counterparts. Due to these special properties, NMs have vast range of uses in the areas of electronics, medical, agriculture and many others which has increased interest in the nanotechnology sectors [1]. Among the NMs, some major varieties are metals, polymers, and semiconductor metal oxides (SMOs) [2]. Because of its versatile uses and expanding study, metals and SMOs are regarded as the richest nanomaterial families [3,4], among all these varieties. Due to their potential oxidation strength, high light stability, and non-toxicity, the different SMOs nanomaterial have different applications such as sensors, actuators, capacitors, medicinal sciences, environmental sciences, solar cells, Li-ion batteries, and others [5,6]. Among SMOs, barium oxide (BaO) NMs is particularly useful because of its unique properties and versatile applications. Wide bandgap n-type semiconductor barium oxide (BaO) is both conductive and semiconductor in nature [7]. It can be used in the pharmaceutical industry [8], electrical energy generation [4], self-cleaning [4], sensors and actuators [9], and catalysts [10] etc. There are many techniques for nanoparticles (NPs) synthesis of BaO, including precipitation, sonochemical, solid-state, hydrothermal, sol-gel, microwave, supersaturated reverse micelles and microemulsion process [11]. But a major problem for both present and future medicinal and bioengineering uses, particularly those that are in vivo, is the hazardous effects of these methods. This necessitates the use of the more green, simple, and non-hazardous biogenic/biosynthesis process as an alternative for the synthesis of BaO NPs. Using extracts of the green parts of different plants those are abundant in alkaloid, flavonoid, phenol, tannin, and amino acid phytochemicals as a substance that reduces for the synthesis of more stable BaO NPs [1]. The NPs produced by biogenic are simple, environmentally safe, biocompatible, non-toxic, and economically advantageous [12].

Additionally, certain post-synthesis procedures have been modified as a method for enhancing nano metal oxide characteristics and also to check the impacts of this treatment on them. For instance, it's firmly established that properties of metal oxides microstructural and morphological are significantly altered with the use of extremely powerful ionizing radiation, such as X-rays, gamma photon, and particle radiation in space, nuclear environments, and other settings. This radiation also produces a wide spectrum of defects, which can alter the optical, sensing and electrical features of the materials [[13], [14], [15]]. Gamma (γ) radiation which can be produced in the lab from the radionuclides Co-60 and Cs-137, is an electromagnetic (EM) waves with high energy is strongly ionizing and reacting with semiconducting materials causes ionizing and/or atomic shifts, which cause imperfections in crystals such as displacements, holes and flaw in clusters [16]. The crystalline defects in the crystal lattice resulting from the absorbed doses of the material modify the optical and structural features of the materials, as well as the overall functionality of devices manufactured with SMOs [[17], [18], [19]]. Research on the effects of gamma rays on the physical properties of SMOs and further materials has been conducted extensively, such as α-MoO_3_ [16], ZnO nano-powder [17], WoO_3_ NPs [20], sol-gel syn. h-MoO_3_ nanorods [21], spray-pyrolysis syn. h-MoO_3_ nanorods [22] etc. In this current research, we feature an easy and green technique to synthesize BaO NPs. As far as we know, no prior research has been conducted on how γ-irradiation affects the physical characteristics of green synthesized BaO NPs using *Moringa Oleifera* leaves. Therefore, the novelty and significance of our present experiment is to investigate for the first time, how various total absorbed doses of γ-radiation affect the morphological, optical, and structural characteristics of green synthesized BaO NPs.

Hence, synthesis of BaO NPs using *Moringa Oleifera* leaves and the subsequent ^60^Co - gamma irradiation effects on it at various doses (0, 25, 50, and 75 kGy) are described here in this report to have a greater understanding of how gamma irradiation could affect their structural and optical characteristics. By using XRD, FT-IR spectroscopy, FESEM, and UV–vis–NIR spectrophotometer techniques, the effects of gamma radiation on BaO NPs were assessed.

## 2 Materials and methods

### 2.1 Preparation of plant extracts

From the premises of the Atomic Energy Research Establishment (AERE), fresh and healthy *Moringa Oleifera* leaves were gathered, and the botanist identified the plant. To remove the surface dirt, three cycles of normal tap water washing were followed by a demineralized water wash for the leaves. These well cleansed green leaves were then sun-dried under the sun light for 7 days and grinded into a fine powder. 13 g of powder from *Moringa Oleifera* leaves was added to 300 ml of deionized water, and then the mixture was heated with a magnetic stirrer for 2 h at 60 °C. Whatman filter paper was used to filter the plant extract three times after it was cooled to produce a clear extract solution. At ambient temperature, the pH of the extract solution was 7.3.

### 2.2 Green synthesis of barium oxide (BaO) NPs

**Fig. 1:**
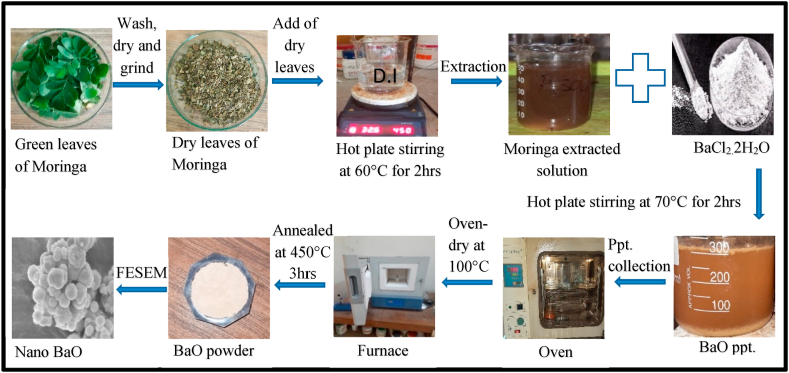
Schematic diagram of green synthesis of BaO from *Moringa Oleifera*.

To make BaO NPs, 300 ml the *Moringa Oleifer* leaves extract solution was transferred to a beaker and 6.0 g antecedent salt BaCl_2_ was added in it. The resultant solution (pH 6.89) was then heated for 2 h at 70 °C. After allowing the precipitate to cool, it was centrifuged three times at 5000 rpm for 30 min. The sample was cleaned and then dried at 100 °C for 2 h in an incubator (K & K Scientific and Medical Equipment's, Korea), then annealed for 3 h at 450 °C in an open-air furnace (KSL1100X, MTI Corporation, China). The resulting samples was identified as BaO NPs and described with the help of variety of techniques. The schematic diagram in Figure-1 depicts a general study layout of this research.

### 2.3 Gamma (γ) irradiation

The green synthesized barium oxide **(**BaO**)** NPs were exposed to radiation with the use of a 33.43 kCi commercial Cobalt-60 gamma irradiation system at the Institute of Food and Radiation Biology (IFRB), Atomic Energy Research Establishment (AERE), Bangladesh Atomic Energy Commission. Before radiation, a demo sample located 11 cm from the source was used for a liquid phase dosimetry (Ceric-cerous system), having a radiation rate that is absorbed of 11.24 kGy/h. To get the absorbed doses of 25 kGy, 50 kGy, and 75 kGy, the BaO NPs were exposed to radiation for various periods of time [23].

### 2.4 Characterizations techniques

Investigations were conducted on the effects of irradiation on the optical, morphological, and structural characteristics of the green synthesized BaO NPs utilizing XRD, FTIR, UV–vis–NIR spectrophotometer technique and FESEM. The thermally annealed sample was analyzed using an X-ray diffractometer (3040-X'Pert PRO, Philips) using CuKα radiation of wavelength 1.54056 Å, where 2θ = 10°–80°, operating voltage = 40 kV, current = 30 mA, and scanning speed = 1° min^−1^. FTIR (Model: FTIR-ATR PerkinElmer Spectrum Two) was used to conduct functional group and phase stability studies for wavenumbers between 2000 and 400 cm-1. FESEM (Model-JSM-6700F, JEOL Ltd., Tokyo, Japan) was utilized to look into the distribution of particle sizes and morphological properties of BaO NPs. The UV–Vis–NIR spectroscopy data (Model: PerkinElmer UV–Vis_NIR spectrometer Lambda 1050) data were used to calculate the optical bandgap using the reflected spectra in the 200–800 nm wavelength range.

## 3 Result and discussion

### 3.1 Structural analysis

**Fig. 2:**
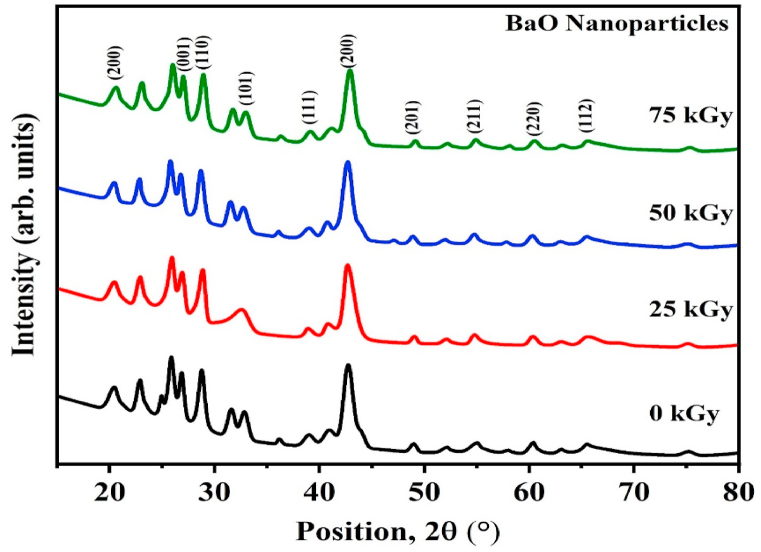
X-ray diffraction patterns of pure and gamma irradiated BaO (25, 50, and 75 kGy) NPs.

XRD analyses of pure and gamma-irradiated samples were conducted to examine the effects of γ-irradiation on the crystalline structure of green synthesized barium oxide (BaO) NPs. The results are displayed in Fig. 2. The diffraction peak for pristine BaO sample with (200), (001), (110), (101), (111), (200), (201), (211), (220) and (112) planes, demonstrate the poly-crystalline nature of the sample and confirmed the tetragonal structure of BaO. The diffracted pattern pure and the samples exposed to gamma radiation are well-aligned with the standard JCPDs card no-026-0178 [7,22]. According to the XRD patterns, the synthesized BaO NPs do not contain any further phases or diffraction peaks corresponding to intermediary reactant impurities. The number of recognizable diffracted peaks after gamma irradiation on pure BaO remains unchanged, although the peak intensity and position have changed significantly. The prominent diffraction peak observed at (200) crystal plane at the diffracted angle of 42.76° suggests the preferential orientation for pristine BaO sample. For 25 kGy and 50 kGy, the prominent peak (200) position are 42.68° and 42.70°, respectively. The preferred orientation (200) moved towards a smaller diffraction angle, 2θ, compared to the pure BaO sample that had lesser absorbed doses of 25 kGy and 50 kGy. Moreover, the position of prominent peak (200) of irradiated BaO is 42.92° for 75 kGy, which moved towards a higher diffraction angle, 2θ respect to pristine BaO sample as seen in Fig. 2. Strains in the lattice framework and changing inter-planar distance values are the causes of this shift phenomenon [24]. Fig. 2 illustrates how the intensity of the (200) peak gradually declined as the overall absorbed dose of gamma-rays increased. This may be the result of restructuring or crystal defects in the BaO crystal structure. The scattering factor, which is related to the number of electrons per atom in the crystal lattice, determines the peak intensity [25]. Nonetheless, ionization induced on by gamma radiation generates some amount of electrons. In addition [26], clarified that peak intensity change is caused by a number of other parameters, including temperature, polarization, absorption, Lorentz, and crystal structure alteration. It follows that every one of these variables might be involved in the fluctuation of XRD peak intensity. The decline in peak intensity signifies a decline in crystallinity [27]. Numerous studies reported that when the overall absorbed dose of gamma radiation increases, the point defect caused by oxygen vacancies causes a decrease in crystallinity [14,28,29]. The micro-structural details and various structural data are revealed by the XRD data. The investigation of XRD patterns can be applied to ascertain the effects of gamma photon irradiation regarding the stacking fault (*ε*), dislocation density (δ), lattice strain (*ε*), crystallite size (D), inter planar distance (d), and lattice constants (a, b, and c).

The lattice constants “a", “b", and “c" of the tetragonal structure of BaO may be computed using the inter-planer distance d of the h k l plane using the miller indices h, k, and l by the following equation no. 1 [30].

(1) $$\frac{1}{d_{hkl}^{2}}=\frac{h^{2}}{a^{2}}+\frac{k^{2}}{b^{2}}+\frac{l^{2}}{c^{2}}$$

Here, $d_{hkl}$ is the inter-planer distance of the plane {h k l}. For tetragonal phase a = b ≠c and α = β = γ = 90°, The values of inter-planer space $d_{hkl}$ of BaO NPs were determined by using the following Bragg's equation no. 2 [31].

(2) $$2d_{hkl}\;\sin\;{ѳ}_{hkl}=n\lambda$$

In this case, “n" denotes the order of diffraction (often n = 1), "λ" represents the Cu Kα1 radiation's X-ray wavelength (λ = 1.5406 Å), and "ѳ_hkl_" is the Brags diffraction angle. The *hkl* and $d_{hkl}$ values for the pristine BaO is well matched with the results observed in the JCPDS card-026-0178. The calculated values of lattice parameters of pristine BaO were a = b = 4.22 Å and c = 3.11 Å for tetragonal phase which correspond well with standard results (a = b = 4.31 Å and c = 3.20 Å) and previous reported values [7,9]. It was seen, lattice parameters vary randomly with the changes of gamma photon radiation. The changes in the lattice parameters with the variation of radiation are the consequence of a structural change induced on by exposure to gamma radiation [20]. The parameters are also close to the standard values and there are almost no significant changes shown in the gamma irradiated BaO samples. The values of lattice parameters of pure and gamma irradiated BaO NPs are displayed in Table 1.

**Table-1:** Lattice parameters of pristine (0 kGy) and gamma irradiated (25, 50, and 75 kGy) BaO NPs.

Sample (kGy)	Lattice parameters		
	a (Å)	b (Å)	c (Å)
0	4.867	4.867	3.31
25	4.885	4.885	3.32
50	4.888	4.888	3.33
75	4.867	4.867	3.31

From the broadening of XRD peaks, the D-S and W–H techniques are most commonly used to estimate the lattice strain, dislocation density, and crystallite size [32]. The estimated crystallite size (D) of the constructively scattered region normal to the reflecting planes is used by D-S's method by the equation no. 3 [21],

(3) $$D=\frac{K\lambda }{\beta_{hkl}\;\cos\;\theta_{hkl}}$$

Where λ is the wavelength used (1.54 Å), K denotes the shape factor having constant value of 0.9, $\beta_{hkl}$ expresses the intensity of full with half maximum (FWHM) in radians and θ is the Bragg's angle.

Crystallographic defects or dislocations interrupt the regular patterns of a crystal structure. Other dislocations in the sample impede the movement of the dislocation. Therefore, a higher hardness is implied by a higher dislocation density. The following formula no. 4 can be employed to evaluate the dislocation density [16],

(4) $$\delta =\frac{1}{D^{2}}$$

where “δ” denotes the dislocation density and “D” is the crystallite size, respectively.

A measure of the distribution of lattice constants caused by structural defects such lattice dislocations is known as lattice strain. These atoms in the crystals are what cause crystal defects because they are displaced from reference-lattice locations [33]. Equation (5) was used to compute the lattice strain (*ε*) [16],

(5) $$\varepsilon_{hkl}=\frac{\beta_{hkl}}{4\;\tan\;\theta_{hkl}}$$

Where $\varepsilon_{hkl}$ denotes the lattice strain and $\beta_{hkl}$ the full width half maximum (FWHM) in radians and θ is Bragg's angle.

The disorder of a crystallographic plane is characterized by the stacking fault, a particular kind of crystal defect. It is therefore considered to be a planar defects. The equation no. 6 was employed to determine the stacking faults [34].

(6) $$SF=\left[\;\frac{2\pi^{2}}{45{\left(\;3\;\tan\;\theta \right)}^{\frac{1}{2}}}\right]\beta_{hkl}$$

Where $\beta_{hkl}$ denotes the full with half maximum (FWHM).

The W–H method is used to determine crystallographic characteristics with more accuracy [35]. The most commonly used approach that considers size broadening ($\beta_{D}$) and strain broadening ($\beta_{S}$) is the W–H approach. Simply adding $\beta_{D}$ and ${\;\beta }_{S}$ in equation no. 7 yields the measured line width.

(7) $$\beta_{hkl}=\beta_{d}+\beta_{s}$$

with the values of βd and βs from Equations (3), (5) substituted, will obtain equation no. 8,

(8) $$\beta_{hkl}=\frac{k\lambda }{D\;\cos\;\theta }+4\varepsilon \;\tan\;\theta$$

By rearranging equation (8), will get equation no. 9,

(9) $$\beta_{hkl}\;\cos\;\theta =\frac{k\lambda }{D_{WH}}+4\varepsilon_{WH}\;\sin\;\theta$$

**Fig. 3:**
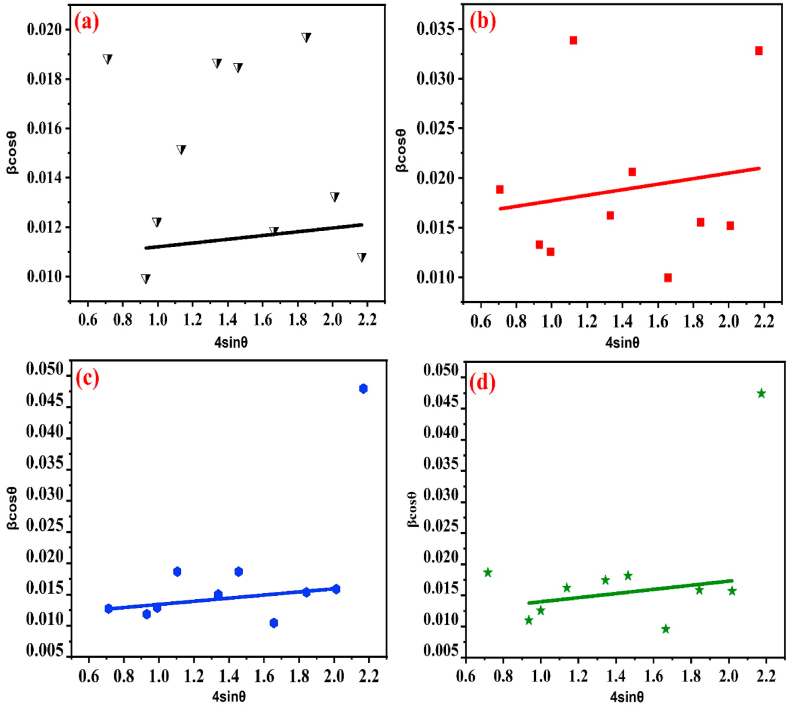
W–H plots of (a) pristine, (b) 25 kGy, (c) 50 kGy, and (d) 75 kGy gamma irradiated BaO NPs.

W–H equation is this which is shown above. The uniform deformation model (UDM) of W–H is represented by equation (9) which takes the shape of a straight line with the formula y = mx + c. In this model, strain is considered to be uniform in all crystallographic directions [36]. As shown in Fig. 3(a)–(d), from the slope and y-intercept, we may calculate strain (***ε***_ℎ*kl*_) and crystallite size, respectively according to Eq. (9).

(10) $$C.I=\frac{{A.\;}_{\text{cr}.\text{phase}\;}}{{A.}_{\text{total}}}\times 100\%$$

Using formula no. 10, the crystallinity degree was determined as the ratio of the crystalline phase area to the total area under the XRD curve,

Here, the crystallinity degree is denoted by C·I, the corresponding crystalline phase area is represented by A. cr.phase, and the total area under the XRD pattern is displayed by A. total.

**Fig. 4:**
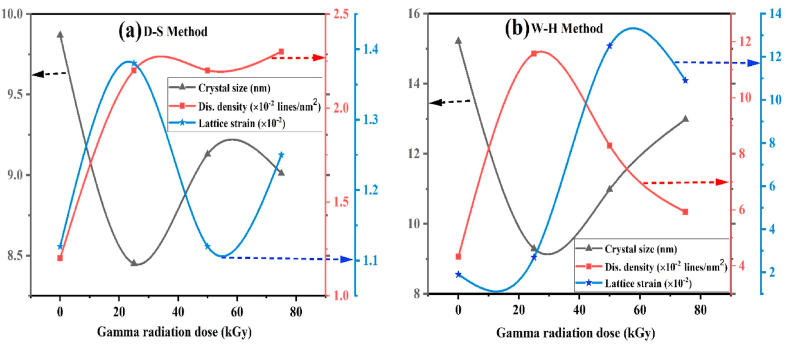
Variation of strain, dislocation density, and crystallite size of pristine and gamma irradiated BaO NPs with changes the gamma radiation dose using (a) D–S, and (b) W–H methods.

Variation of different parameters of pristine and gamma irradiated BaO samples regarding both techniques are shown in Table 2 and Fig. 4(a)–(b) provides a graphical depiction of them. From the table, considering all the relevant peaks the crystallite sizes (D) calculated by D–S equation are 9.87, 8.45, 9.13, and 9.01 nm for 0, 25, 50, and 75 kGy gamma irradiated BaO NPs, respectively. It is important to note that the corresponding strain and dislocation density values exhibit a reversal trend in relation to the D-S formula. When comparing pristine to 25 kGy irradiation BaO samples, the crystallite size decreases, a degradation of crystallinity is seen, based on the calculated crystallite size measurements obtained using the W–H and D-S techniques. The size of the crystallites are decreasing, which may be caused by their splitting into smaller ones. However, at high absorbed doses (50 and 75 kGy) in both D-S and W–H methods, grain boundary collapse-induced coalescence of the synthesis of large-sized crystallites appears to be triggered by tiny crystallites, hence increasing the average crystallite size. Crystallinity at high doses not showed a systematic trend, but overall it decreased with increasing the doses [37]. At dose 25 kGy, the lattice strain increases from 1.12 × 10^−2^ to 1.38 × 10^−2^ probably as a result of some grain boundaries breaking down and crystallite sizes decreasing. Overall the trend of lattice strain is random. With increasing absorbed doses, the dislocation density initially increases at 25 kGy from 4.32 × 10–5 to 11.57 × 10–5, indicating an increase in crystallographic imperfections and defects in the prepared BaO NPs. The trend is also similar for the higher doses. Although there are differences in the use of D-S and W–H techniques to examine the behavior of strain fluctuation, dislocation density, and crystallite size variation in pristine and gamma-irradiated BaO NPs, but these close differences are also similar to values mentioned in Ref. [22].

**Table 2:** Structural parameters of pristine (0 kGy) and irradiated (25, 50, and 75 kGy) BaO NPs.

Sample (kGy)	D-S method			W–H method			Staking factor, SF	Crystallinity (%)
	Lattice strain, ε $\times 10^{-2}$	Crystallite size, D (nm)	Dislocation density, δ (lines/nm^2^) $\times 10^{-2}$	Lattice strain, ε $\times 10^{-5}$	Crystallite size, D (nm)	Dislocation density, δ (lines/A^2^) $\times 10^{-5}$		
0	1.12	9.87	1.2	1.9	15.21	4.32	0.9872	85.00
25	1.38	8.45	2.2	2.7	9.29	11.57	0.9835	84.96
50	1.12	9.13	2.2	12.5	10.98	8.29	0.9835	75.92
75	1.25	9.01	2.3	10.9	12.98	5.92	0.9834	82.62

Dislocation density provides more details about the quantity of defects in crystals found in the materials, and lattice strain provides an assessment of the lattice constant distribution based on by dislocations, oxygen vacancies, and particle size. It is significant to notice that in both the D-S and W–H formulas, the corresponding dislocation density and strain values follow in reverse order. The decrement trend of stacking fault suggests that the BaO NPs have crystal defects. The trend in crystallinity was similar to that in crystallite size. Crystallinity and crystallite size are two major factors which are commonly regarded as full width at half maximum (FWHM) and intensity of the XRD peaks.

### 3.2 Functional group analysis by FTIR

**Fig. 5:**
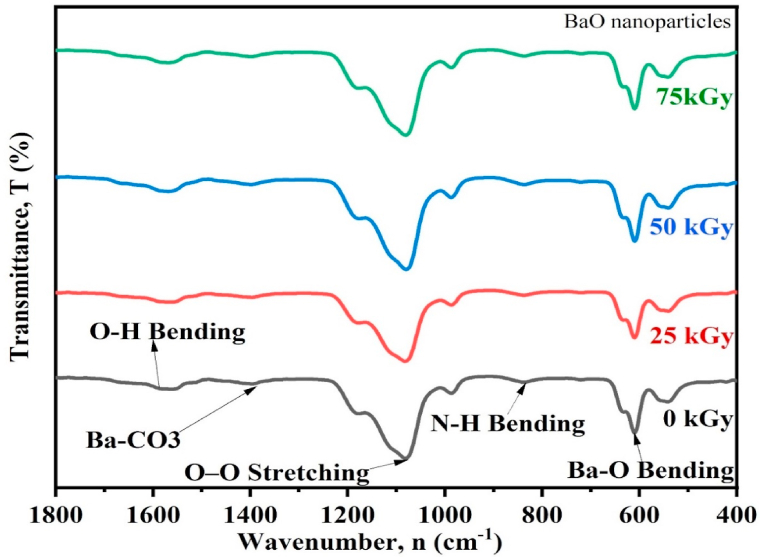
FTIR of pristine (0 kGy) and gamma irradiated (25, 50, and 75 kGy) BaO NPs.

The functional groups present in the pristine (0 kGy) and gamma irradiated (25, 50, and 75 kGy) BaO samples were examined ranging from 4000 to 400 cm^−1^and displayed in Fig. 5. In the FTIR band of inorganic compounds, the band position, relative intensity and bandwidth rely on its particles size and morphology. And it may be changed with the changes of irradiation dose because of the significant impacts of irradiation dose on particles the size and morphology of the compound [16]. A characteristic of O–H groups of adsorbed surface water, a vibrational peak around 1580 cm^−1^ was observed, after dose variation which shifted to 1586 cm^−1^ [38]^.^ A noteworthy change is seen within hydrogen bonds O–H vibration following a change in gamma absorption dose. The vibration of the OH group rises with γ-irradiation demonstrates how H–*O*–H bonds are getting weaker and longer [21]. The peak originated at around 1400 cm^−1^ is perhaps connected to the formation of barium carbonate, which comes from BaO-NPs absorbing ambient CO_2_ [9]. After dose variation which shifted to 1406 cm^−1^, The Ba–CO_3_ bond vibration increases with γ-irritability show that the Ba–CO_3_ bonds are getting longer and weaker. A peak observed at around 1076 cm−1 is linked to O–O stretching vibration modes [9]. After dose variation which changed to 1080 cm^−1^, as γ-irritation rises, the O–O stretching modes of vibration also increase, revealing the weakening and lengthening of O–O bonds. A high transmitted peak was observed at 610 cm^−1^ for Ba–O vibration, which shifted to 614 cm^−1^with the variation of dose.

It is possible that the little local heat generation induced by the variation in irradiation dose absorption is what is causing the defect creation, as evidenced by the little variation in the transmitted peak strength and location of the bending vibrations of the Ba–O bond. The assignments of all the identified bands in the spectrum are revealed in Table 3.

**Table 3:** FTIR Spectroscopy data of pristine (0 kGy) and gamma irradiated (25, 50, and 75 kGy) BaO NPs.

Functional group	Wavenumber (cm^−1^)				
	0 kGy	25 kGy	50 kGy	75 kGy	Ref. value
O–H bending vibration	1580	1582	1586	1586	1630 [4]
Ba–CO_3_ vibration	1400	1406	1406	1406	1455 [9]
O–O stretching vibration	1076	1078	1078	1080	1059 [9]
Ba–O stretching vibration	610	612	614	610	615 [9]

### 3.3 Surface morphological study

**Fig. 6:**
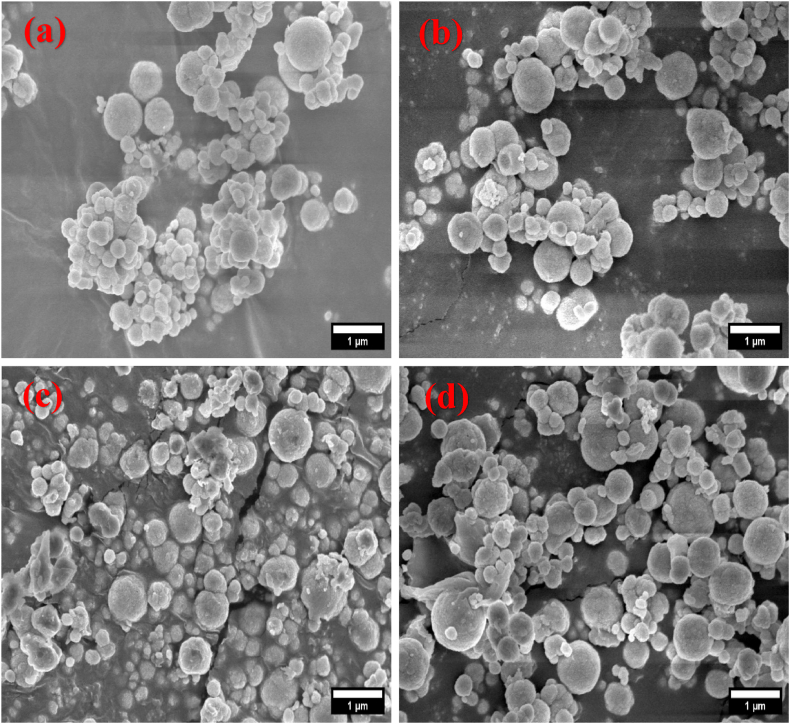
FESEM images of (a) pristine, (b) 25 kGy, (c) 50 kGy, and (d) 75 kGy gamma irradiated BaO NPs.

**Fig. 7:**
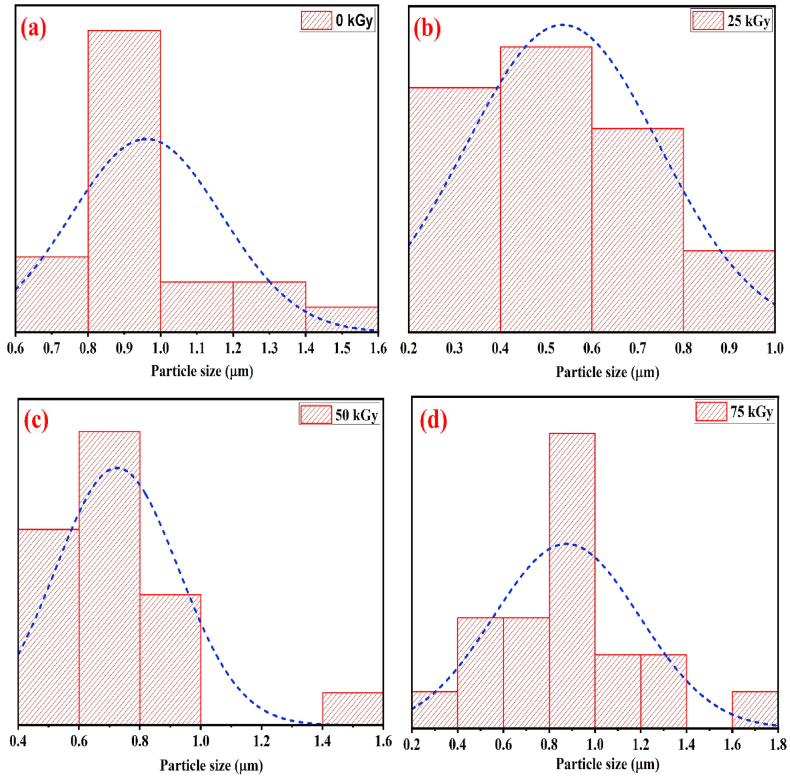
Grain distribution of (a) pristine, (b) 25 kGy, (c) 50 kGy, and (d) 75 kGy gamma irradiated BaO NPs.

The FESEM images ( × 10,000) and their related histograms of particle size distribution of pure and all gamma-irradiated BaO NPs are displayed in Fig. 6(a)-(d) and Fig. 7(a)-(d), respectively.

As shown in FESEM images, the surface of the BaO NPs was clearly disrupted, with many randomly oriented islets displaying a rough surface appearance. After gamma irradiation on BaO NPs, local heat generation causes the grain boundaries to collapse, which leads to grain agglomeration in Fig. 6 (c). The radiation led to a localized heating in the surface atom mobility. The change of the surface morphology of the BaO NPs may have a role in this mobility. Although morphology of the BaO NPs changed with radiation, but the surface did not experience any damage, indicating that the small amount of surface stress due to radiation was insignificant. Grain size on average of pristine BaO NPs calculated using the histogram is 0.960 μm. However, when exposed to γ-radiation, the grain size decreases from 0.960 to 0.875 μm during the rise in doses from 0 to 75 kGy. For the as-deposited and gamma-irradiated BaO samples, the X-ray diffraction analysis reasonably agrees with the average crystallite size declining. It is possible to standardize for a wide range of optoelectronic and bio-sensing applications featuring significant porosity and surface roughness nature seen from the FESEM images of BaO NPs [39,40].

### 3.4 Optical properties study

**Fig. 8:**
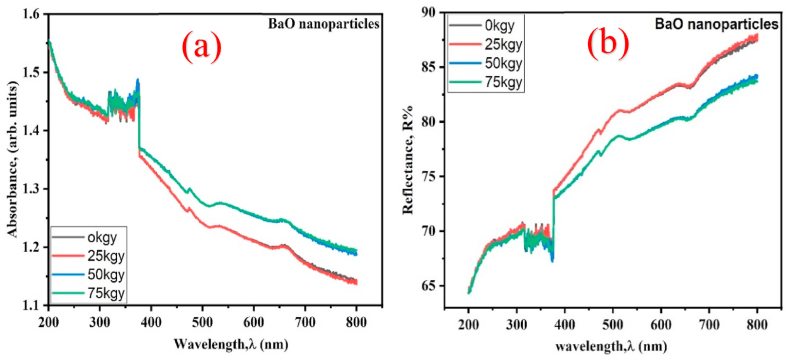
(a) Absorbance and (b) Reflectance spectra of pristine and gamma irradiated BaO NPs.

Using UV–vis (DRS), the optical absorbance and reflectance bands of pure and gamma-irradiated BaO NPs viewed at the wavelength ranging from 200 to 800 nm at room temperature are displayed in Fig. 8(a)–(b). The graph **8(b)** indicates that the diffuse reflectance spectra begin to increase at 25 kGy and gradually decrease at absorption doses of 50 kGy and 75 kGy. This suggests that while crystallinity of BaO was imperfect for additional increases in gamma absorption dose at 50 and 75 kGy, the stoichiometry was improved at the initial lower dose at 25 kGy. Graph **8(a)** shows the respective absorbance spectra of pure.

and gamma irradiated BaO NPs depending on the incident UV–Vis light wavelength.

For assessing the sort and estimate of the optical energy spectrum gap (Eg) of crystal and amorphous in semiconducting materials, a well-established technique that assesses the optical absorption at the fundamental absorption band edge can be applied. The E_g_ values of pure and gamma-irradiated BaO samples may be estimated by applying equation no. 11 of the well-known K-M function based on optical absorption [41].

(11) $${\{(F\left(R\right)*h\upsilon )\}}^{\frac{1}{n}}=A\left(h\upsilon -E_{g}\right)$$

where n is a constant that defines the kind of transition, A = the proportionality constant, hv = photon energy, F(R) = absorption co-efficient, and Eg = the optical band gap value.

**Fig. 9:**
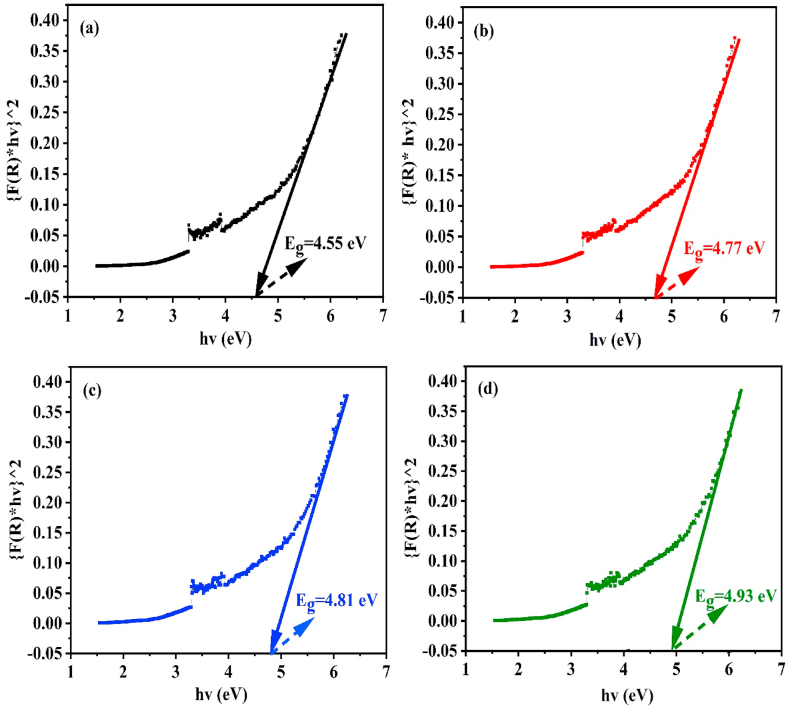
Plot for optical band gap measurement of (a) pure, (b) 25 kGy, (c) 50 kGy, and (d) 75 kGy gamma irradiated BaO NPs.

The BaO samples exhibit a direct type optical bandgap, as shown by the presence of a straight line in the spectra. The factor n, whose value is dependent upon the transition modes, characterizes the optical transition process. The direct allowed, indirect allowed, direct forbidden, and indirect forbidden modes have n values of 1/2, 2, 3/2, and 3, respectively [30]. As displayed in Fig. 9(a–d), the direct bandgap (E_g_) of the BaO NPs before and after radiation exposure can be calculated using a plot of {F(R)*hv}^2 as a function of hv. The values of E_g_ for BaO are estimated by the intercept of {(F(R)*hv)}^2 on the energy axis (x-axis).

In Fig. 9(a)–(d), it is shown that the pristine and 25, 50, and 75 kGy gamma irradiated BaO NPs have bandgap energies (E_g_) of 4.55, 4.77, 4.81, and 4.93 eV, respectively (Table 4). In comparison to the bulk phase of BaO, which has a bandgap energy of 4.4 eV, the pure and gamma irradiated BaO Nps have a bandgap energy greater than that of BaO in bulk phase and consistent with the previous literatures [4,7,9,42,43]. We see that the optical band gap progressively rises from 4.55 to 4.93 eV with the increase in absorbed doses from 0 to 75 kGy, demonstrating the blue shift trend of band gap changes. It is useful for windows made of solar cells as well as other optoelectronic and photonic devices [44]. The impact of quantum size and the up-shifting trend of band gap due to rising absorbed doses may be primarily caused by an increase of defects and/or structural disorders (ionizing effect, displacement effect, and structure alteration caused defects) subsequent to the interaction of gamma radiation with materials. These defects and/or structural disorder in NPs produce state localization (density of defect states), that widen the optical band gap [[45], [46], [47]]. Rise in the E_g_ values with increasing the γ-doses may be caused by the reduction in crystallinity and the removal of vacant oxygen ions in BaO as a result of γ-irradiation [48]. This assumption is in line with the findings of the XRD analysis. Additionally, the absorption edge moving to higher energies and the widening of the value of E_g_ in BaO are caused by energy splitting between the valence and conduction bands changing, along with an increase in electron concentration resulting from a rise in the Fermi level of conduction band [27,49]. This experimental finding indicates that the optical bandwidth is altered and crystallite size is also impacted by the irradiation.

**Table 4:** Optical band gap data of pristine (0 kGy) and gamma irradiated (25, 50, and 75 kGy) BaO NPs.

Samples (kGy)	Eg (eV)	Eg (eV) of Bulk phase BaO	Ref. values Eg (eV)
0	4.55	4.4 [42]	4.65,3.82,4.33,3.70 [4,7,9,43]
25	4.77		
50	4.81		
75	4.93		

## 4 Conclusion

Here, we demonstrated how gamma irradiation affects the physical properties of barium oxide (BaO) NPs green synthesized from *Moringa Oleifera* leaves and exposed to radiation at doses of 0, 25, 50, and 75 kGy of Co-60 gamma radiation. These impacts were investigated using XRD, FTIR, FESEM and UV–Vis techniques. The XRD characterization explored that the crystallite size of the pristine nanostructured BaO decreased with the increment in dose as determined by the D-S method. The crystallites size was estimated to be 9.87, 8.45, 9.13, and 9.01 nm for 0, 25, 50, and 75 kGy irradiated samples, respectively. The W–H method provided further evidence in favor of this decreasing trend in crystallite size. In case of dislocation density and lattice strain in both D-S and W–H methods follow in reverse trend that of crystallite size. In FTIR, for pristine BaO sample the major vibrational and stretching peaks were seen at 610, 1076, 1400, and 1580 cm^−1^ for Ba–O, O–O, Ba–CO_3,_ and O–H groups, respectively. For the gamma radiated BaO samples these peaks for functional groups remain same but peaks shift at a higher position when the absorbed dose of gamma radiation increases. The FESEM micrographs showed the spherical shape of the pristine BaO sample and gamma irradiation did not result in any noticeable changes. In UV–Vis, with an increase in γ-dose from 0 to 75 kGy, the irradiation causes the optical band gap to rise from 4.55 to 4.93 eV, which is attributed to the loss of crystallinity, the addition of crystal defects, the rise in disorder, and the addition of lattice strain. The findings demonstrated that defects in nanostructured BaO and influenced their optical, thermal, morphological, and structural characteristics. Despite its subsequent defects BaO NPs shown higher gamma radiation stability and absorption intensity. These results showed that nanostructured BaO would be appropriate for a range of adverse conditions applications, such as space exploration and nuclear reactors.

## Data availability statement

Data will be made available on request.

## CRediT authorship contribution statement

**Md Rabiul Islam:** Writing – original draft. **Sapan Kumar Sen:** Writing – review & editing, Conceptualization. **Arup Kumar:** Investigation. **M.S. Islam:** Investigation. **Md. Serajum Manir:** Conceptualization. **Zannath Ara:** Writing – original draft. **M.D. Hossain:** Writing – review & editing. **M.K. Alam:** Investigation.

## Declaration of competing interest

The authors declare that they have no known competing financial interests or personal relationships that could have appeared to influence the work reported in this paper.
